# Beware of ligand efficiency (LE): understanding LE data in modeling structure-activity and structure-economy relationships

**DOI:** 10.1186/s13321-017-0236-9

**Published:** 2017-09-11

**Authors:** Jaroslaw Polanski, Aleksandra Tkocz, Urszula Kucia

**Affiliations:** Institute of Chemistry, University of Silesia, 9 Szkolna Street, 40-006 Katowice, Poland

**Keywords:** Ligand efficiency, Ligand-target binding affinity, Fragmental metrics, Weight metrics, Molar metrics, Avogadro number, Big data

## Abstract

**Background:**

On the one hand, ligand efficiency (LE) and the binding efficiency index (BEI), which are binding properties (B) averaged versus the heavy atom count (HAC: LE) or molecular weight (MW: BEI), have recently been declared a novel universal tool for drug design. On the other hand, questions have been raised about the mathematical validity of the LE approach.

**Results:**

In fact, neither the critics nor the advocates are precise enough to provide a generally understandable and accepted chemistry of the LE metrics. In particular, this refers to the puzzle of the LE trends for small and large molecules. In this paper, we explain the chemistry and mathematics of the LE type of data. Because LE is a weight metrics related to binding per gram, its hyperbolic decrease with an increasing number of heavy atoms can be easily understood by its 1/MW dependency. Accordingly, we analyzed how this influences the LE trends for ligand-target binding, economic big data or molecular descriptor data. In particular, we compared the trends for the thermodynamic ∆G data of a series of ligands that interact with 14 different target classes, which were extracted from the *BindingDB* database with the market prices of a commercial compound library of ca. 2.5 mln synthetic building blocks.

**Conclusions:**

An interpretation of LE and BEI that clearly explains the observed trends for these parameters are presented here for the first time. Accordingly, we show that the main misunderstanding of the chemical meaning of the BEI and LE parameters is their interpretation as molecular descriptors that are connected with a single molecule, while binding is a statistical effect in which a population of ligands limits the formation of ligand-receptor complexes. Therefore, LE (BEI) should not be interpreted as a molecular (physicochemical) descriptor that is connected with a single molecule but as a property (binding per gram). Accordingly, the puzzle of the surprising behavior of LE is explained by the 1/MW dependency. This effect clearly explains the hyperbolic LE trend not as a real increase in binding potency but as a physical limitation due to the different population of ligands with different MWs in a 1 g sample available for the formation of ligand-receptor complexes.

**Graphical abstract:**
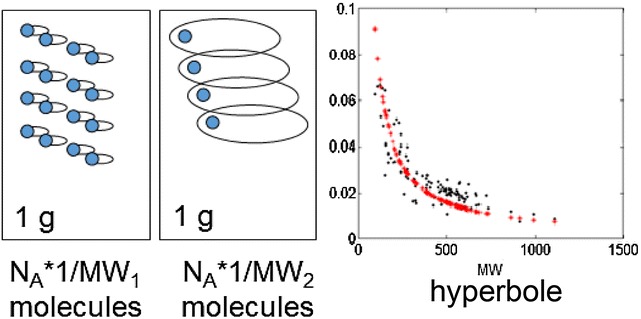
.

## Background

Ligand efficiency (LE), which is the free energy of binding (B) averaged versus the heavy atom count (HAC), has recently been declared to be a novel universal tool for drug design that will permit the substantial optimization of both molecular mass and lipophilicity *by ameliorating the inflation of these properties that has been observed in current medicinal chemistry practice and to increase the quality of drug candidates* [1]. As this effect may have resulted from the application of current procedures that are based on the common use of IC_50_ or binding affinity (K_i_) in drug optimization, the efficiency approach appears to be especially attractive in controlling both the molar binding potency and HAC or molecular weight (MW) at the same time.

However, questions have recently been raised about the mathematical validity of the definition of LE [2–4]. Specifically, for example, as indicated by the anonymous reviewer of the first version of this publication: *LE, whether derived from deltaG, K*_*d*_ *, K*_*i*_ *, or IC*_*50*_ *, has an arbitrary 1* *M concentration unit built into it. Changing this unit will, in general, result in a change in the perception of efficiency. Therefore, the dependence of LE, e.g. derived from deltaG, on the concentration used to define the standard state implies that LE derived from deltaG is thermodynamically meaningless.* However, despite the fact that although the con arguments were basically introduced as early as 2009 [4], the situation is still not clear, just to cite a recent discussion. In the Ref. [5] we read: *LE […] widely used to normalize potency for size, does not, in fact, normalize potency for size. LE decreases and appears to plateau as size, or number of heavy atoms, increases. Several plausible hypotheses were proposed to explain this observation in terms of ligand flexibility and/or entropic penalties, reduced surface area available for interaction, target specific restrictions and size*-*dependent complexity that reduced the probability of optimal fitting*. The answer requires a *simple example of fuel efficiency* to justify the *mathematics* of LE trends [6]. In fact, neither the critics nor the advocates were precise enough to provide any understandable mathematics or chemistry of the LE metrics. Moreover, because these parameters are in common use as early estimators for drug optimization, apparently the numerous con arguments appeared to be not convincing enough for the drug design audience. The reason is that this allowed for the understanding of the mathematical or chemical inaccuracies; however, they have never cleared the reason for the observed puzzle of the LE (BEI) hyperbolic trend explicitly enough. Because the trend obviously indicates an advantage in binding efficiency for the small molecules, which is both attractive and expected in drug design, the LE (BEI) approach has become more and more popular in academia and pharma drug design.

Herein, we show that the basis for the LE (BEI) puzzle can be cleared up by a careful analysis of its chemical meaning. Accordingly, we demonstrated that the main misunderstanding of the chemical meaning of the BEI and LE parameters is their interpretation as molecular descriptors that are connected with a single molecule, whereas binding is a statistical effect in which a population of ligands is an important factor that determines the pairing of ligand-receptor complexes. Therefore, LE (BEI) should not be interpreted as molecular (physicochemical) descriptors (binding per Dalton) but as statistical properties (BEI—binding per gram, where a gram is a mole of Daltons); (LE—binding per a mole of HAC). Accordingly, the puzzle of the surprising behavior of LE is explained by the 1/MW dependency of a 1 g substance of molecules that have the MW, which is a property that can be measured. This effect clearly explains the hyperbolic LE trend not as the real increase in binding potency but by the availability of ligands for the ligand-receptor complexes. Additionally, we analyzed the LE-type intensive parameters including a broad spectrum of both molecular descriptors and properties. We demonstrated that individual LE trends can be explained by the basic rules of chemistry, thereby indicating how important it is to distinguish between molecular descriptors and properties [7, 8]. More specifically, we compared two datasets. The first was the experimental binding thermodynamics for approximately 100 protein–ligand complexes [9]. The second was the big data of the market prices of a large commercial library of building blocks [10].

## Methods

### Molecular descriptors versus properties

Basically, chemical compounds, i.e. both molecules and substances, can be represented by molecular descriptors, i.e. indicators that relate to the molecule or molecular structure that can be calculated from a molecular representation or by the properties that are to be measured experimentally if there are real values or that require predictions during molecular design [7]. However, it is not always easy to distinguish between these two data types. Let us analyze molecular weight (MW). It can be a property when measured for molecules, e.g. in MS spectrometry or even when we are weighting a mole, i.e. the Avogadro number of the molecules or its fraction, but alternatively it can also be a descriptor when we are estimating the MW of a single molecule simply by summing the atomic mass contributions to the total MW. The weight of 1 mol of a substance will be its MW (g/mol), while the weight of a single molecule will be its MW (Da). The correlation between these two variables is 100% and creates a major trick in chemistry when we are mapping substances to molecules and vice versa. In fact, we need an Avogadro number (N_A_), which is a chemical routine, for this transformation that is generally overlooked. Therefore, MW (Da) * N_A_ = MW (g/mol).

### LE definitions and metrics

Formal definitions of ligand and binding efficiency LE and BEI have previously been described in the literature and various forms of these parameters were precisely described by Cortes-Ciriano recently [11]. We will interpret these parameters in their widest sense as given below:

1 $${\text{LE}} = {\text{binding property}}/{\text{HAC}}$$

where the binding property is any property that is measured in order to define the interactions between a ligand and a receptor and HAC is the heavy (nonhydrogen) atom count.

1a $${\text{BEI}} = {\text{binding property}}/{\text{MW}}$$

where the binding property is any property that is measured in order to define the interactions between a ligand and a receptor and MW corresponds to the molecular weight in Daltons (Da).

Generally, in the literature both LE and BEI are interpreted as molecular descriptors in the sense of physicochemical descriptors, for BEI compare especially Cortes-Ciriano [11] and Abad-Zapatero et al. [12]. In the context of the differentiation of the molecular descriptors and properties discussed in previous paragraph, these are (binding) properties per molecular fragment defined by the MW (Da) or the HAC (number of atoms). It has been completely overlooked, that alternatively, BEI can be interpreted directly as a property, the direct measure of the binding of a 1 g sample of a substance. Accordingly, LE is a property—a direct measure of the binding of a mole of HAC.

LE was originally developed in order to compare the maximal ligand-target affinity [13] including small, nonhydrogen ligand cations or anions. This, in turn, determined that non-hydrogen atom count, namely HAC, was the natural normalizing measure. However, the atom count (AC), hydrogen count or molecular weight can generate analog metrics. On the other hand, we can substantially broaden the LE definition to include any property (P) or molecular descriptor (MD). Thus, defining the efficiency parameter (PE or MDE), which for a property normalized versus HAC (as for a standard LE) will be defined by:

2 $${\text{PE}}_{\text{HAC}} = {\text{P}}_{\text{mol}} /{\text{HAC}}$$

where P_mol_ relates to a molar-normalized property, e.g. molar-binding affinity.

### Data sets

The thermodynamic ∆G data of a series of 102 protein–ligand complexes that interact with 14 different target classes were assembled by gathering bioactivity information from the *BindingDB* database [9] by Reynolds and Holloway.

The catalog data for a commercial compound library of ca. 2.5 mln synthetic building blocks were downloaded from the internet site (http://www.abamachem.net/). This large library includes 2,248,243 chemicals that are offered on the market [10]. The database contains easily accessible information that can be downloaded in the SDF format. The records were carefully inspected before further processing, e.g. duplicated notations were removed.

Calculations were performed using the KNIME Analytics Platform (version 3) on an Intel Core 2 Duo CPU 1.80 GHz computer system with 4.00 GB RAM and a 64-bit Windows 10 operating system. Instant JChem version 14.7.28.0, which was released in 2014, and additional self-programmed scripts were used for structure database management. Graphs were plotted using MATLAB version R2015b.

## Results and discussion

In practice, LE is much more popular in drug design than BEI. Equation 2, which is the definition of LE, can be converted to:

3 $${\text{PE}}_{\text{HAC}} = {\text{P}}_{\text{mol}} /{\text{MW}}*{\text{MW}}/{\text{HAC}}$$

where P_mol_ relates to a molar-normalized property, e.g. molar-binding affinity.

**Fig. 1:**
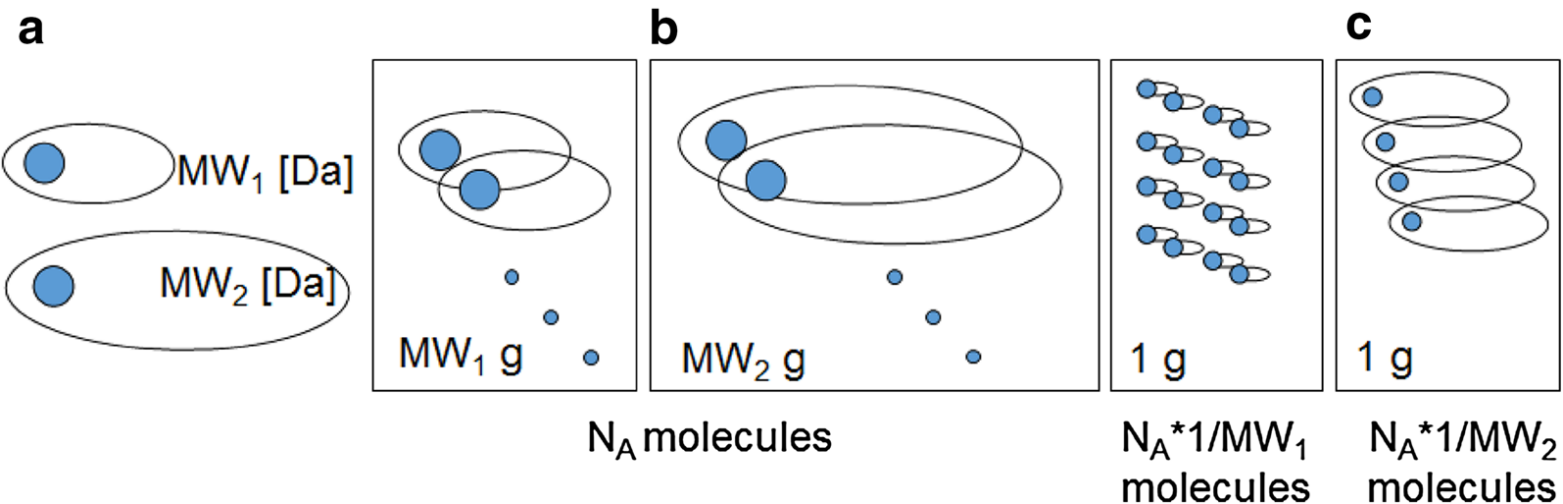
Avogadro statistics—two molecules, MW_1_ (Da) and MW_2_ (Da), **a** can be scaled to a substance and can agglomerate N_A_ molecules using mole metrics. The weight of the substances will be MW_1_ (g/mol) and MW_2_ (g/mol), respectively (**b**). Alternatively, mapping **c** can be performed in order to maintain a steady substance weight, e.g. of 1 g, and then the number of molecules will be different for two molecules each time and will have a value of 1/MW_1_ and 1/MW_2_, respectively. A virtual 1 (Da) fragment is indicated in blue

Equation 3 allows us to precisely understand the chemical sense of LE mapping. Accordingly, PE_HAC_ is the interaction of two terms. The first is P_mol_/MW, i.e. P_mol_ normalized by MW. This term defines also BEI which is a possible LE alternative [12]. A second term MW/HAC rescales BEI into the HAC dimension. To properly understand the operations described by Eqs. 2 and 3, we explained the fragmental (Fig. 1a), molar (Fig. 1b) and weight (Fig. 1c) metrics that are used to map atomic molecules (Fig. 1a) into a real substances (Fig. 1b, c) in chemistry. In order to calculate LE, we use fragmental metric (Fig. 1a) in an attempt to calculate the share of binding for a single Da (HAC fragment) in a single molecule (Fig. 1a) indicated in blue in Fig. 1. Mapping molecules to a mole of a substance, a mole metric (Fig. 1b), will preserve the same number of molecules (N_A_), but the weight of the samples of 1 mol will differ and amount to MW_1_ and MW_2_ grams, respectively. Alternatively, mapping by weight metric will preserve the constant weight, e.g., 1 g. A surprising feature of the latter method of mapping is that the difference in a number of molecules in 1 g will be given by the numbers of N_A_ * 1/MW_1_ and N_A_ * 1/MW_2_, respectively. A surprise here comes from the fact that we are simply not accustomed to this metric. Although, we do not realize this fact, the weight metric is often used in medicinal chemistry, e.g. when measuring binding affinity or IC_50_ (P_gram_), we test the weighted samples (g) in order to obtain the P_gram_ in (kcal/g) and eventually, at the very end, recalculating the P_gram_ into the P_mol_ scale.

In turn, because binding affinities are given in the literature as P_mol_, therefore, P_gram_ can be calculated from the simple equation P_gram_ = P_mol_/MW, which essentially looks the same as the first term in Eq. 3, however, fragmental descriptor refers to a single molecule, while binding affinity refers to a substance. Although formally we need the Avogadro number N_A_ to relate the measured affinity and the affinity of a single molecule (or single fragment), we can do without N_A_ because the MW (Da) (molecular descriptor) and the mole of a substance MW (g/mol) (a property) are the same values.

The most interesting features of the metrics in Fig. 1 are that:

1. All three measures in Fig. 1 preserve a steady invariable size of the 1 Da fragment
2. Unlike the molar measure (P_mol_), the weight metric, LE (BEI), does not have a thermodynamic meaning because the concentration of ligands is not normalized for the molecules of different sizes and
3. The multiplier, which scales a molecule to a constant weight is proportional to 1/MW.

Generally, LE (BEI) is interpreted in the literature as a *molecular physicochemical descriptor* that relates to a single molecule. This appears to be the main misunderstanding because both the concentration and the binding are statistical properties that are related to molecular populations. Accordingly, LE (BEI) are also properties. The concentration here determines a population of the ligands that are available for the receptor to form ligand-receptor complexes, while P_mol_ determines binding potency.

### The trend of binding efficiency versus molecular descriptor and economic price

**Fig. 2:**
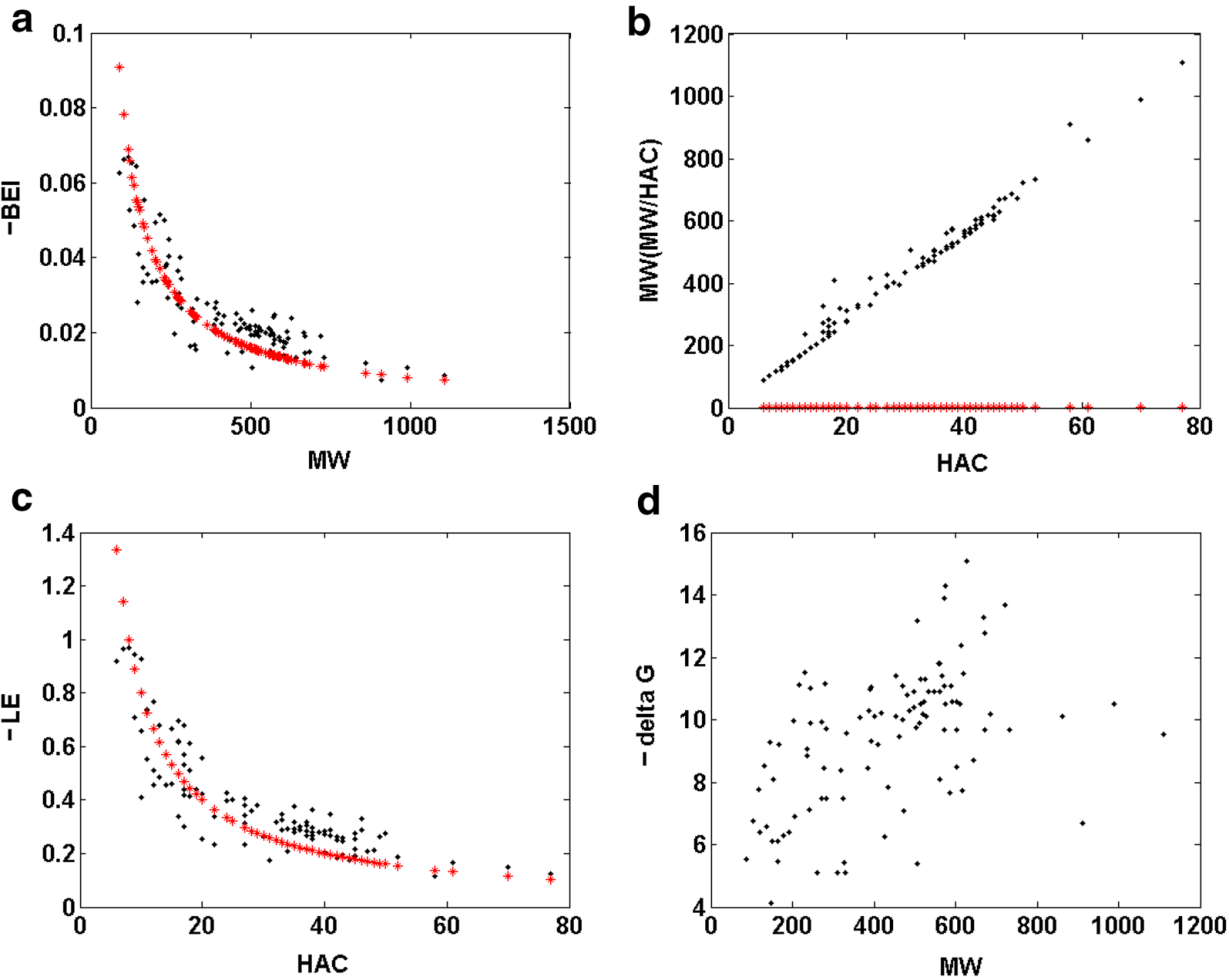
The binding ΔG values plotted versus MW or HAC—**a** BEI versus MW (black) versus a hyperbole 8 * 1/MW (red), **c** LE versus HAC (black) versus hyperbole 8 * 1/HAC (red), **d** delta G versus MW compared to **b** MW versus HAC (black) or MW/HAC versus HAC (red), data according to Ref. [9]; hyperboles plotted without optimization

In Fig. 2a, c we plotted the BEI and LE_HAC_ for the thermodynamic ∆G data of a series of ligands that interact with 14 different target classes, which were extracted from the *BindingDB* database by Reynolds and Holloway [9], respectively. We can see that a hyperbole approximates the data for BEI. The large differentiation of the targets means that deviations from the model can be observed; however, the trend is obvious.

In order to become more familiar with the LE type metrics in Table 1, we analyzed the impact of the individual terms of Eq. 3 to illustrate their chemical meaning and mathematics. Therefore, Eq. 3 can be decomposed into Eq. 3a:

3a $${\text{PE}}_{\text{HAC}} = {\text{P}}_{\text{mol}} * \, 1/{\text{MW}}*{\text{MW}}/{\text{HAC}}$$

**Table 1:**
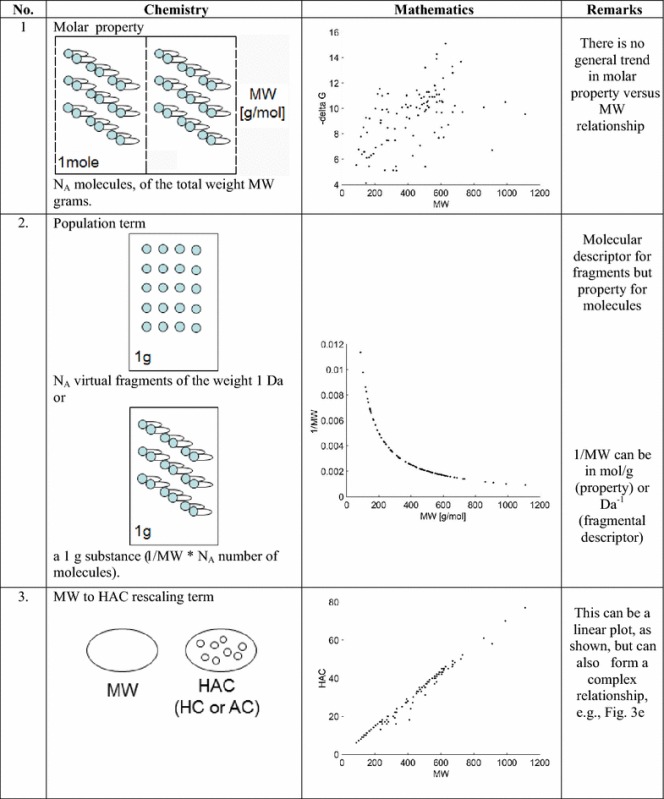
Chemical meaning and mathematical function of the individual terms that define LE (Eq. 3a)

Data according to Ref. [9]

Accordingly, PE_HAC_ is now the interaction of three terms. The first term is molar property, second is 1/MW—a number of molecules in a 1 g sample of molecules of the weight MW (Da) (a population term) and a third term, the MW to HAC rescaling factor. Essentially, the population term 1/MW dominates the real molar property P_mol_ and the MW to HAC rescaling factor is more or less a constant value as MW versus HAC has a close to linear relationship for the thermodynamic ∆G data analyzed in this publication. Therefore, the population term 1/MW and not a real property explains the trend of LE, thereby solving the puzzle of LE behavior. This determines a significant increase of LE for a small MW and a plateau for a high MW.

We could conclude at this point that the chemistry of LE behavior is trivial. In our opinion it is not the case, and it is not a coincidence that LE has been misinterpreted in significant articles in high impact journals (PNAS [13], Nature Reviews Drugs Discovery [1]). We can better understand the origins of this delusion by probing the LE models for nonbinding parameters.

**Fig. 3:**
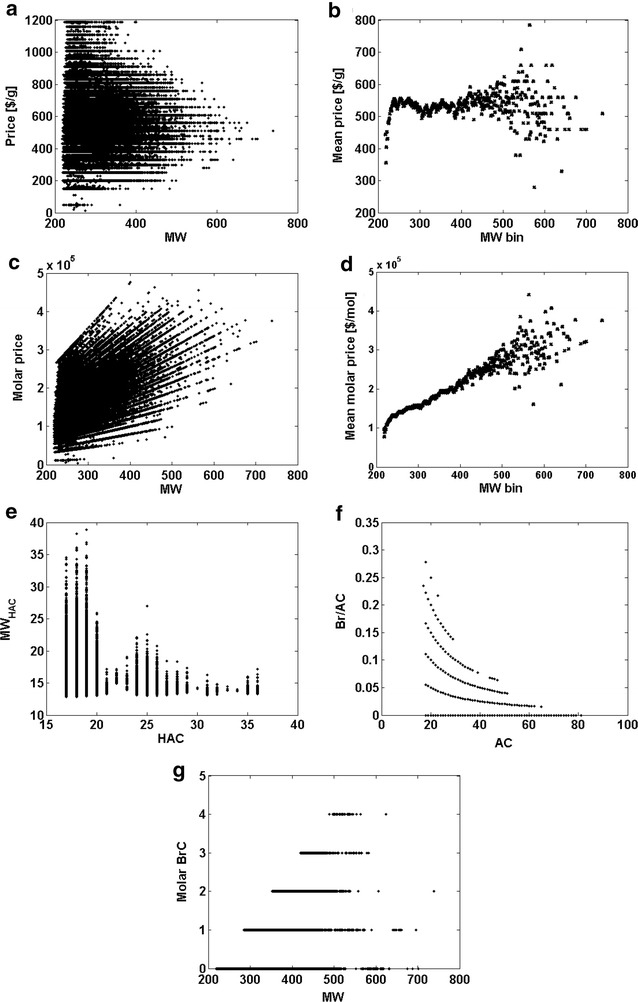
Selected descriptors of the ca. 2.5 mln library data for chemical compounds according to Ref. [10]. **a**–**d** Market prices plotted versus MW **a** weight price ($/g) versus MW, **b** binned statistics of mean price ($/g) versus MW bin, **c** molar price ($/mol) versus MW, **d** binned statistics of mean molar price ($/mol) versus MW bin. Selected efficiency descriptors (**e**–**g**): **e** MW/HAC versus HAC, **f** Br/AC versus AC, **g** molar BrC versus MW

First, problem is to identify such models. The relationship between a chemical structure and its physical or chemical properties is an essential concept in chemistry and this method is an important decision-making guide, for example, in drug design. In fact however, it is the market that eventually decides the success of any pharmaceutical; therefore; we need economic considerations to fully understand a fate of a drug. Economic behavior, in particular, a price of a drug is an example of the nonbinding parameter important for molecular design. Is there any relationship between a chemical structure of a drug and its economic potential? On the one hand, explaining economic effects is an extremely complex issue. On the other hand little market data is available for drugs. Accordingly, the problem remains unexplored. In contrast, to drugs a variety of economic data are available for other chemicals. Therefore, in Fig. 3a–d, we present a structure-economy analysis for a commercial compound library of ca. 2.5 mln synthetic building blocks [10]. It is critical to note that the price, which is an economic property, is typically listed in the catalogs of chemical compounds in $/g (Fig. 3a) and not $/mol (Fig. 3c), which means the efficiency scale is standard in economics. The relationships observed in Figs 3a, c determines market behavior of a large quantity of chemicals. This decides that instead of a single model a bunch of linear plots can be identified in Fig. 3c relating molar prices to MW, while weight prices (Fig. 3a) form a series of horizontal plots, i.e., within each individual plot weight price does not depend upon MW (Fig. 3a). To further investigate the price data in Fig. 3b, d, we illustrated their MW binned statistics. This indicates that in economics the price of a sample normalized by weight is on average unvarying across a large range of MWs, while mean molar price forms a linear plot versus MW. Accordingly, with a decreasing MW, on average, we can get a larger number of molecules at the same price. Instead, if normalized to the molar metric, the same fragments are cheaper at smaller MWs. Interestingly, even now the plot of the mean price (the LE type parameter) versus MW indicates a drop of prices at the low MWs, which can be interpreted as a hyperbolic like trend (Fig. 3b). However, this effect can be observed only within the lowest MW range, despite the fact that the molar and weight metrics are in a similar mathematical relation as the binding properties, i.e. the price $/g is given by the interaction of the molar price and 1/MW. Mean molar price is more or less a linear function of MW (Fig. 3d) or in other words, an increase of MW also means an increase of the weight of a sample to be paid. The larger the quantity of weight, the larger is also the price, which is one of the essential rules of economics. Accordingly, a comparison of the binding versus economic LE data type illustrates that the understanding of important chemical effect is required to understand the LE trends versus molecular size. While the stoichiometry of ligand-target pairing limits the binding LE, the macroscopic weight determines the price. Unlike binding, pricing is not a statistical property; therefore, hypothetically, the price can tag a single molecule.

Recently, ratio type descriptors have been used more frequently in drug design [14]. This includes parameters such as ratio of O/(N + O) [14] or the number of molecular fragments, e.g. a fraction of sp3 carbons to all of the carbon atoms [15]. Therefore, in Fig. 3e–g, we present several molecular descriptors which show the molar and weight normalization, respectively, and was calculated for a large chemical compound library of ca. 2.5 mln chemical compounds. The first is a simple example, i.e. MW/HAC versus HAC (Fig. 3e). In particular, we can observe that MW versus HAC does not always have a linear relationship. In turn, Fig. 3f shows a plot of the number of arbitrarily selected atoms, e.g., bromines (Br count; BrC), that was normalized by the atom count (AC), i.e. the BrC_AC_ (Br/AC) within this library. The individual hyperboles map the molecules that have the same number of Br atoms. A question now arises of whether we can identify any chemical property that scales acc. to the weight metric. The answer is positive because we can discover that a simple analytical attribute of the percentage content follows this metric. In turn, if we probe a relationship of bromine count BrC versus MW, then, BrC will take an integer value indicating bromine atoms in a single molecule, which obeys a molar scale rule as is shown in Fig. 3g. Molecular descriptors and properties of the efficiency type can support us in illustrating various chemical and pharmaceutical effects. The nonlinearity of the hyperbolic population term is of potential interest in modeling in pharma and chemistry. However, this requires a complete understanding of the metrics that are used and the chemical effects that determine these metrics.

## Conclusion

In conclusion, in this paper, the chemical meaning and mathematical form of ligand efficiency (LE) type data is explained. Therefore, LE is related to binding per gram (property) while the puzzle of the surprising behavior of LE is explained by the 1/MW dependency of the weight metric. We analyzed how this influences the weight-normalized data for economic and molecular property and descriptor data.

## Abbreviations

MW: molecular weight
HAC: heavy atom count
AC: atom count
BrC: bromine count
LE: ligand efficiency; the indexes, e.g. MW or HAC refer to the scaling factor, e.g. MW or HAC
BEI: binding efficiency index
P: property, where *mole* or *gram* indexes refer to the molar or weight metrics, respectively
N_A_: Avogadro number
